# An investigation of integrating the finite element method (FEM) with grey system theory for geotechnical problems

**DOI:** 10.1371/journal.pone.0270400

**Published:** 2022-06-24

**Authors:** Yao Li, Guowei Zhu, Qingchao Zhang

**Affiliations:** 1 School of Geosciences and Surveying Engineering, China University of Mining and Technology-Beijing, Beijing, P. R. China; 2 State Key Laboratory Coal Resources and Safe Mining, China University of Mining and Technology-Beijing, Beijing, P. R. China; 3 School of Earth Science and Engineering, Hebei University of Engineering, Handan, Hebei, P. R. China; University of Vigo, SPAIN

## Abstract

Numerical simulation is very important to solve geotechnical problems. However, it is difficult to obtain required comprehensive and accurate information such as parameters, boundary conditions, and etc. In this paper, a grey distributed parameter model, which integrates the finite element method (FEM) with the grey system theory, was proposed to address the issue. The analysis of grey properties on rock and soil system was performed. The equilibrium equations, geometric equations, physics equations and related differential equations were obtained, each of the equations contains grey parameters and variables. And the discretization and solution methods of the FEM with the grey variables were discussed. An example of deep-buried circular mining tunnel was applied to test the proposed model. The calculation results were compared with those of the exact solution (analytical solution) and the classical FEM, respectively, through which the rationality of the proposed model was demonstrated. For the first time, grey variables and grey parameters are defined in geotechnical numerical simulation. The expressions of basic equations with grey variables are given respectively. A grey distributed parameter model which integrates the FEM with the grey system theory is proposed to solve geotechnical problems, and the optimal solution to the proposed model is determined through calculation and comparison of an application example. The proposed numerical model with grey variables not only has the advantage of grey system theory, but also greatly improves the adaptability and application effect of the model, which contributes to the prediction and evaluation problems in geological engineering, geotechnical engineering, water conservancy engineering and civil engineering with complex structures.

## 1. Introduction

Geotechnical engineering solves problems in stability of surrounding rock in various underground chambers (such as tunnels, building foundations, goaf, and etc.) and also in the formation and change of various geological disasters on earth’s surface. The empirical formula method, engineering analogy method and numerical simulation method are used for solving geotechnical problems nowadays, among which the numerical simulation provides evaluation results of surrounding rock stability more scientifically. However, these methods are based on a ‘white’ (a well-defined) system. In fact, the structure of geotechnical system is complicated, and most is in the blind area of people’s vision. Data of rock and soil system obtained by various engineering exploration methods are limited and partial, and it makes the geotechnical system a non-essential grey system which could be better studied with grey system theory [1].

In the field of geotechnical engineering, grey system methods have been actively applied and some achievements have been made. LU and Rosenbaum [2] predicted future ground movement based on geotechnical properties and historical behavior with artificial neural networks (ANN) and grey system theory. Jing et al. [3] established a simulation experiment based on the grey system theory with the actual measurement data of the studied surface. The vertical displacement of the surface was used as a reference array, and a comparison array with four groups from the experiment constituted a grey relative analysis system. The relationship between different mining subsidence and vertical displacement of the surface was determined, which provided a theoretical basis for the layout of the mine work area and the reduction of surface building damage. Bachir et al. [4] applied grey system to predict working face pressure, with particle swarm optimization (PSO) algorithm obtaining optimal parameters in the grey model, as a result, achieving good prediction effect. Deng et al. [5] applied the grey system theory on predicting crown settlement of Jiulianshan tunnel, and compared it with the traditional regression analysis method. Lei et al. [6] established the classification table of surrounding rock stability according to the geological data. On the basis of grey relational analysis and fuzzy recognition principle, the objective function was constructed by the least square method. The relational analysis of five indexes affecting the stability of surrounding rock was carried out, and the grey optimal classification model to evaluate surrounding rock stability was established. Tasci and Kose [7] used multivariable grey models GM (0, N) and GM (1, N) to determine the deformation on the crest of the Keban Dam in Turkey. Miao et al. [8] used the Verhulst-Grey model to calculate the parameters of the landslide time forecast model established on the basis of displacement monitoring data. Zhang et al. [9] proposed a model which combined the optimized grey discrete Verhulst model with back propagation (BP) neural network to better predict foundation pit settlement. Zhang et al. [10] updated the traditional grey model (TGM) (1, 1) to the TGM (1, 1, p, q) grey model by adding a nonlinear time correction factor and optimizing the weighting coefficient of the background value. Wang et al. [11] proposed a grey neural network model based on the grey theory with the neural network algorithm, and analyzed the roof compaction data of the return air working face in a mine in Xuzhou. It not only eliminated the shortcomings of neural network model, but also made up for the lack of self-feedback adjustment of grey network. MATLAB was used to simulate and test the roof pressure prediction results of the model, and the results were compared with those of single grey model and single BP neural network model. As can be seen from the work cited above, the application of grey system theory in geotechnical problems mostly employed grey models with lumped parameters which can only predict and evaluate the studied object as a whole, making the simulation of any point with the methods not satisfying.

Moreover, the classical numerical simulation method with distributed parameters is capable of simulating the state of any point. It was used for analysis of the stress, displacement and failure of underground engineering, slope and dam since 1960s and has made great progress with the development of computer technology. Due to the difficulty in obtaining sufficient and comprehensive data such as mechanical parameters, loads and boundary conditions, the application of the classical numerical simulation method is also limited. Therefore, studies on integrating the FEM with uncertainty methods are getting intensive. Kleiber and Hien [12] proposed a new stochastic FEM based on the perturbation method to analyze the structural response. Ghanem and Spanos [13] have proved that the spectral stochastic finite element method could forecast a variety of uncertainties in calculating system responses. Griffiths and Fenton [14] used a random FEM (RFEM) for stochastic analysis of slopes by considering a strength reduction technique. Chaudhuri and Sekhar [15] have adopted the stochastic FEM based on perturbation technique to analyze the flow and transport in a three-dimensional porous formation. Ahmed [16, 17] conducted a stochastic seepage analysis through earth dams and under water-retaining structures by FEM. Zheng et al. [18] used fuzzy FEM to analyze the impact of cold wave on face slab cracking of a concrete-faced rockfill dam (CFRD). A simplified procedure based on FEM with the random field theory was developed by Hongwei Huang et al. [19], for analyzing the longitudinal performance of shield tunnels considering the longitudinal variation of geotechnical parameters. Johari and Talebi [20] considered the uncertainties of soil parameters leads to an uncertain wetting branch of the soil-water retention curve (SWRC), and made a computer program to determine the reliability indices of unsaturated slopes under different rainfall intensities and seepage flow using random FEM (RFEM). Chen and Liu [21] developed a stochastic FEM to solve the calculation precision deficiency caused by spatial variability of dam compaction quality. Liu and Wang [22] proposed a probabilistic method for simulating the entire process of rainfall-induced landslides considering spatial variability of soil properties. A two-stage method containing FEM and material point method (MPM), both with hydro-mechanical coupling, is proposed to simulate a landslide from triggering by rainfall to post-failure large deformation of soils. It can be seen from the work cited above, that FEM is integrated with many uncertainty methods, such as fuzzy-FEM, random-FEM and stochastic-FEM. However, there are few researches on integrating FEM with grey system theory, even though grey system has the advantages of solving system problems in poor information situation.

In summary, up to now, studies of the grey system theory and methods in the field of geotechnical engineering mostly employed grey models with lumped parameters, and the simulation results are lack of accuracy especially when performing analysis of any point. In terms of the classical method (analytical or the numerical) with distributed parameters, although it can be used for simulation of any point, the parameters and other information involved are treated as deterministic, making the response obtained also deterministic. But the actual geotechnical system is not completely this way, and grey system has the advantages of solving system problems with poor and uncertain information. Therefore, in this paper, a grey distributed parameter model (numerical model) which integrates the FEM with grey system theory was proposed to study geotechnical problems, and it can be used to simulate displacement of any point without deterministic input information. The grey properties of rock and soil system are analyzed; various equations with grey variables are established including mechanical equilibrium equations, geometric equations, physics equations, and related differential equations. Then, the discretization and solution method of the finite element equation with grey variables is discussed. The proposed model (FEM with grey variables) was used to analyze an example of deep-buried circular tunnel, and the calculation results were compared with those of the exact solution (analytical solution) and the classical FEM, respectively. The uniqueness, objectivity and rationality of the application of the FEM with grey variables in geotechnical problems are demonstrated.

## 2. Grey property of rock and soil system: A non-essential grey system

Using mathematical models to describe the rock and soil system is challenging, because mathematical models need detailed original information. However, the actual rock and soil system is complex, and full access to its information is limited based on current engineering technology and equipment. Therefore, the rock and soil system is a non-essential grey system. The grey property of rock and soil system is mainly reflected in the following aspects:

### 2.1 Complexity of rock and soil mass

Because the rock and soil system is a natural geological body meaning that its spatial distribution varies with crustal tectonic movement and metamorphism, the rock and soil system is heterogeneous. The stress field of rock and soil includes gravity stress, tectonic stress, seepage stress and temperature stress. There are many factors affecting the stress field, such as lithology, faults, groundwater, temperature, and etc. These factors are extremely irregular due to the heterogeneity and randomness of geological bodies. Therefore, the stress field is complex, and the boundary conditions of the studied area are difficult to determine.

### 2.2 The limited access to raw data

Due to the limitations of manpower, time, cost and field conditions, only partial geotechnical information can be obtained. The structure of rock and soil system is very complex, and most of them are in the blind area of people’s sight. The data of geotechnical system collected by various engineering exploration methods are always limited and partial. The understanding of this actual system is incomplete, and it makes the rock and soil system a non-essential grey system.

## 3. Definition and operation properties of grey number

### 3.1 Definition of grey number

Grey number (GN) is the most fundamental concept of grey system, a basic unit of grey system and grey mathematics.

Definition 3.1 [23].

Define P(c) as a connotation-cognition problem that can be quantified and I(c) as the cognition-information field of P(c). R is the field of real number and D is a subset of R. Suppose that ⊗ is an uncertain number about P(c), *d*^*o*^ is the only potential true number of the uncertain number ⊗ and the acquiescing number $\tilde{⊗}$, and ⊗ satisfies the following condition,

$$\forall \tilde{⊗}\;\text{of}⊗\Rightarrow \tilde{⊗}\in D(c)$$

where D(c)$=\left\{\tilde{⊗}\left|\tilde{⊗}\right.\right.$ comes from I(c) and *d*°approaches I(c); *d*^*o*^, $\tilde{⊗}\in D$; *d*^**•**^ is the true number of P(c); if *d*^**•**^ occurs, then *d*^*o*^ = *d*^**•**^ holds, and ⊗ and $\tilde{⊗}$ vanish at the same time}, then

1. ⊗ is the grey number about the connotation-cognition problem P(c);
2. D is the number-covered set of ⊗;
3. $\tilde{⊗}$ is the whitened number of ⊗;
4. I(c) is the information background of ⊗;
5. ⊗, D, $\tilde{⊗}$, and *d*^*o*^ are unified by the grey-number element of P(c).

### 3.2 Operation properties of grey number

The operation between grey numbers is usually the operation between number-covered set when engineering information is inadequate.

Definition 3.2 [23].

Suppose that ⊗_*i*_ and ⊗_*j*_ are two continuous grey numbers (CGN), and their number-covered sets are *D*_*i*_ = [*a*_*i*_, *b*_*i*_], (*a*_*i*_ < *b*_*i*_) and *D*_*j*_ = [*a*_*j*_, *b*_*j*_], (*a*_*j*_ < *b*_*j*_), respectively. Let ⊗_*ij*_ = ⊗_*i*_ ∘ ⊗_*j*_ and *D*_*ij*_ = *D*_*i*_ ∘ *D*_*j*_, where ∘ ∈ {+,−,×, ÷}. Then we have that *D*_*ij*_ is the number-covered set of ⊗_*ij*_ and it is as below:

1. *D*_*ij*_ = *D*_*i*_ + *D*_*j*_ = [*a*_*i*_ **+** *a*_*j*_, *b*_*i*_ + *b*_*j*_];
2. *D*_*ij*_ = *D*_*i*_ + *D*_*j*_ = [*a*_*i*_ **+** *b*_*j*_, *b*_*i*_ + *a*_*j*_];
3. *D*_*ij*_ = *D*_*i*_ + *D*_*j*_ = [min{*a*_*i*_*a*_*j*_, *a*_*i*_*b*_*j*_, *b*_*i*_*a*_*j*_, *b*_*i*_*b*_*j*_}, max{*a*_*i*_*a*_*j*_, *a*_*i*_*b*_*j*_, *b*_*i*_*a*_*j*_, *b*_*i*_*b*_*j*_}];
4. *D*_*ij*_ = *D*_*i*_ ÷ *D*_*j*_ = [min{*a*_*i*_/*a*_*j*_,*a*_*i*_/*b*_*j*_,*b*_*i*_/*a*_*j*_,*b*_*i*_/*b*_*j*_}, max{*a*_*i*_/*a*_*j*_,*a*_*i*_/*b*_*j*_,*b*_*i*_/*a*_*j*_,*b*_*i*_/*b*_*j*_}] where 0 ∉ *D*_*j*_. Δ*D* = [*b* − *a*] is the chaos of ⊗, which represents the uncertainty degree of grey number [24], and is further used to evaluate the quality of the proposed grey models in Section 6.2.

Theorem 3.1 [23].

Supposing that ⊗ is a GN and D is its number-covered set. Let ∘ ∈ {+,−,×, ÷} be a binary operation. If *a* is a real number, then *a*⊗ is GN, and its number-covered set is as below:

$$a\circ D=\left\{a\circ d\left|d\in D\right.\right\},$$

Where 0 ∉ *D*_*j*_ when the operator is “÷” [24].

## 4. Definitions of grey parameters and variables, establishment of basic equations and differential equations with grey variables

Elastic mechanics is an important branch of solid mechanics. It studies the deformation and internal force of elastic objects under the action of external forces and other external factors, and it is also the basis of geotechnical problems. As studying the distributed parameter models, mechanical parameters, boundary conditions and stress conditions are essential.

### 4.1 Definitions of grey parameters and variables

The following analysis started from greying out basic variables and parameters, such as *σ* (stress), *ε* (strain), *u* (displacement), *E* (elastic modulus), *μ* (Poisson’s ratio), because they are the foundation of basic elastic mechanics equations [25].

Grey stress: $⊗\sigma_{ij}=\left[\underline{\sigma_{ij}},\bar{\sigma_{ij}}\right]$, where $\underline{\sigma_{ij}}$ and $\bar{\sigma_{ij}}$ represent the lower and upper bounds of grey stress at any point in space, respectively, *i*, *j* = *x*, *y*, *z*.
Grey strain: $⊗\varepsilon_{ij}=\left[\underline{\varepsilon_{ij}},\bar{\varepsilon_{ij}}\right]$, where $\underline{\varepsilon_{ij}}$ and $\bar{\varepsilon_{ij}}$ represent the lower and upper bounds of grey strain at any point in space, respectively, *i*, *j* = *x*, *y*, *z*.
Grey displacement in x-axis direction: $⊗u_{m}=\left[\underline{u_{m}},\bar{u_{m}}\right]$, where $\underline{u_{m}}$ and $\bar{u_{m}}$ represent the lower and upper bounds of grey displacement at any point in x-axis direction, respectively, *m* = *A*, *B*, A and B are 2 nodes in Fig 2.
Grey displacement in y-axis direction: $⊗v_{m}=\left[\underline{v_{m}},\bar{v_{m}}\right]$, where $\underline{v_{m}}$ and $\bar{v_{m}}$ represent the lower and upper bounds of grey displacement at any point in y-axis direction, respectively, *m* = *A*, *B*, A and B are 2 nodes in Fig 2.
Grey elastic modulus: $⊗E=\left[\underline{E},\bar{E}\right]$, $\underline{E}$ and $\bar{E}$ represent the lower and upper bounds of grey elastic modulus, respectively.
Grey Poisson’s ratio: $⊗\mu =\left[\underline{\mu },\bar{\mu }\right]$, $\underline{\mu }$ and $\bar{\mu }$ represent the lower and upper bounds of grey Poisson’s ratio, respectively.
Grey load: $⊗f_{n}=\left[\underline{f_{n}},\bar{f_{n}}\right]$, $\underline{f_{n}}$ and $\bar{f_{n}}$ represent the lower and upper bounds of grey load, respectively, *n* = *x*, *y*, *z*.

### 4.2 Establishment of differential equations with grey variables

The established differential equations with grey variables below satisfy the basic assumptions of elastic mechanics (continuity, uniformity, isotropy and small deformation) [25], and are used to study the equilibrium and deformation of any element of the elastic body.

#### 4.2.1 The equilibrium equations with grey variables

**Fig 1:**
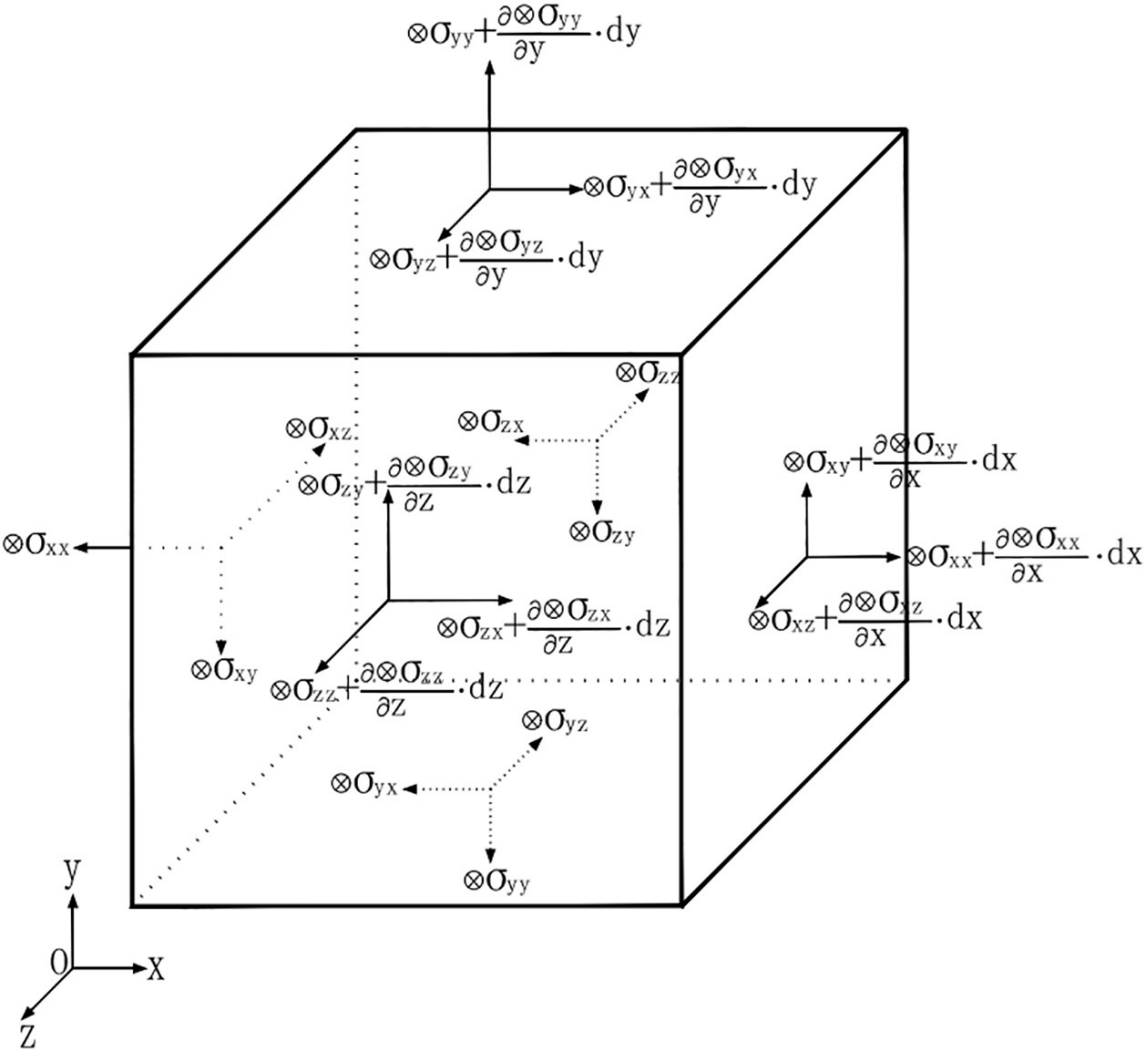
A 3D force equilibrium unit with grey variables.

The equilibrium equations below with grey variables for two-dimension and three-dimension system were derived [25]. As shown in Fig 1, the grey load ⊗*F* was decomposed in the x, y, z direction as (⊗*f*_*x*_ ⊗*F*_*y*_ ⊗*F*_*z*_), according to the force equilibrium:

$$\begin{array}{l} ⊗\sigma_{xx}dydz+⊗\sigma_{yx}dxdz+⊗\sigma_{zx}dxdy=(⊗\sigma_{xx}+\frac{\partial ⊗\sigma_{xx}}{\partial x}dx)dydz+\\ (⊗\sigma_{yx}+\frac{\partial ⊗\sigma_{yx}}{\partial y}dy)dxdz+(⊗\sigma_{zx}+\frac{\partial ⊗\sigma_{zx}}{\partial z}dz)dxdy+⊗f_{x}dxdydz \end{array}$$ (1)

Equilibrium equations with grey variables in x-axis direction can be obtained from Eq (1):

$$\frac{\partial ⊗\sigma_{xx}}{\partial x}+\frac{\partial ⊗\sigma_{yx}}{\partial y}+\frac{\partial ⊗\sigma_{zx}}{\partial z}+⊗f_{x}=0$$ (2)

Similarly, equilibrium equations with grey variables in y-axis and z-axis direction can be obtained, respectively:

$$\frac{\partial ⊗\sigma_{xy}}{\partial x}+\frac{\partial ⊗\sigma_{yy}}{\partial y}+\frac{\partial ⊗\sigma_{zy}}{\partial z}+⊗f_{\text{y}}=0$$ (3)

$$\frac{\partial ⊗\sigma_{xz}}{\partial x}+\frac{\partial ⊗\sigma_{yz}}{\partial y}+\frac{\partial ⊗\sigma_{zz}}{\partial z}+⊗f_{z}=0$$ (4)

For a two-dimension system where the force and deformation of the object in the z-axis direction are ignored, the form of the equilibrium equations with grey variables can be further simplified as:

$$\frac{\partial ⊗\sigma_{xx}}{\partial x}+\frac{\partial ⊗\sigma_{yx}}{\partial y}+⊗f_{x}=0$$ (5)

$$\frac{\partial ⊗\sigma_{xy}}{\partial x}+\frac{\partial ⊗\sigma_{yy}}{\partial y}+⊗f_{y}=0$$ (6)

#### 4.2.2 The geometric equations with grey variables

**Fig 2:**
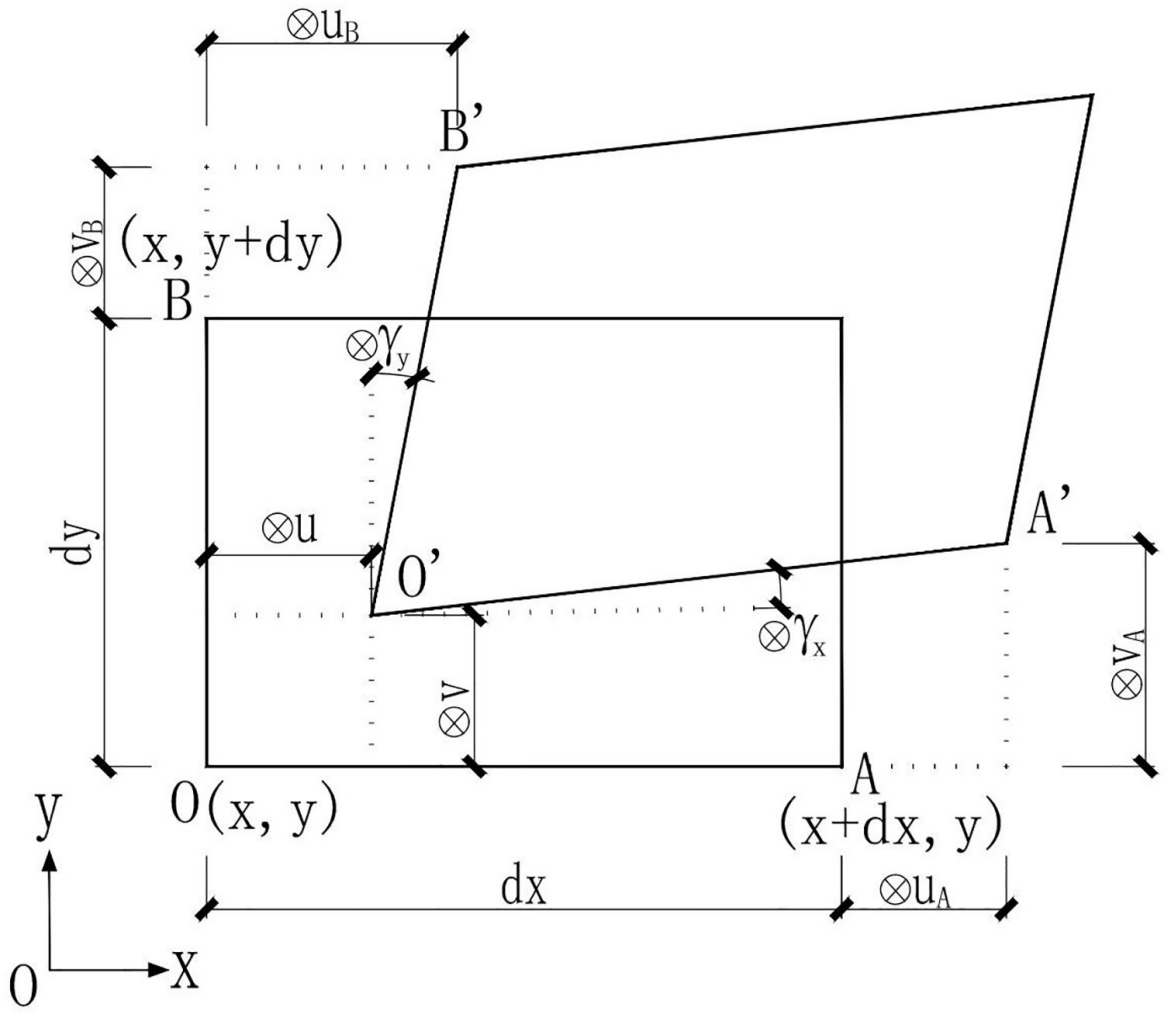
A 2D displacement component with grey variables.

The geometric equations with grey variables are discussed:

The displacement components of the deformable body in the x-axis and y-axis directions are represented by ⊗*u*(*x*, *y*, *z*) and ⊗*v*(*x*, *y*, *z*). By using Taylor’s formula, and ignoring the second-order and above terms and the deformation in the z-axis direction (Fig 2), equations below can be obtained:

$$⊗u_{A}=⊗u(x+dx,y,z)=⊗u+\frac{\partial ⊗u}{\partial x}dx$$ (7)

$$⊗v_{A}=⊗v(x+dx,y,z)=⊗v+\frac{\partial ⊗v}{\partial x}dx$$ (8)

$$⊗u_{B}=⊗u(x,y+dy,z)=⊗u+\frac{\partial ⊗u}{\partial y}dy$$ (9)

$$⊗v_{B}=⊗v(x,y+dy,z)=⊗v+\frac{\partial ⊗v}{\partial y}dy$$ (10)

According to the definition of strain,$⊗\varepsilon_{x}=\frac{O\prime A\prime -OA}{OA}=\frac{(dx+⊗u_{A}-⊗u)/\text{cos}\;\gamma_{x}-dx}{dx}$, small-deformation theory, and Eq (7), it can be obtained:

$$\text{cos}\;\gamma_{x}\approx 1$$

$$⊗\varepsilon_{x}=\frac{O\prime A\prime -OA}{OA}=\frac{(dx+⊗u_{A}-⊗u)/\text{cos}\;\gamma_{x}-dx}{dx}=\frac{⊗u_{A}-⊗u}{dx}=\frac{\partial ⊗u}{\partial x}$$ (11)

Similarly, it can be obtained:

$$⊗\varepsilon_{y}=\frac{O\prime B\prime -OB}{OB}=\frac{(dy+⊗v_{B}-⊗v)/\text{cos}\;\gamma_{y}-dy}{dy}=\frac{⊗v_{B}-⊗v}{dx}=\frac{\partial ⊗v}{\partial y}$$ (12)

According to the small-deformation theory, as the deformation angle is small, ⊗*γ*_*x*_ ≈ tan ⊗*γ*.


$$⊗\gamma_{x}=\frac{⊗v_{A}-⊗v}{dx+⊗u_{A}-⊗u}=\frac{⊗v+\frac{\partial ⊗v}{\partial x}dx-⊗v}{dx+\frac{\partial ⊗u}{\partial x}dx}=\frac{\frac{\partial ⊗v}{\partial x}}{1+\frac{\partial ⊗u}{\partial x}}$$ (13)

Because $⊗\varepsilon_{x}=\frac{\partial ⊗u}{\partial x}\ll 1$, $⊗\varepsilon_{y}=\frac{\partial ⊗v}{\partial y}\ll 1$, it can be obtained:

$$⊗\gamma_{x}=\frac{⊗v_{A}-⊗v}{dx+⊗u_{A}-⊗u}=\frac{⊗v+\frac{\partial ⊗v}{\partial x}dx-⊗v}{dx+\frac{\partial ⊗u}{\partial x}dx}=\frac{\frac{\partial ⊗v}{\partial x}}{1+\frac{\partial ⊗u}{\partial x}}=\frac{\partial ⊗v}{\partial x}$$ (14)

Similarly, it can be obtained:

$$⊗\gamma_{y}=\frac{\partial ⊗u}{\partial y}$$ (15)

Therefore, the grey shear strain is:

$$⊗\gamma_{xy}=\frac{\partial ⊗v}{\partial x}+\frac{\partial ⊗u}{\partial y}$$ (16)

To sum up, the Eqs (11), (12) and (16) are the geometric equations with grey variables of the two-dimension problems. They can be written in matrix form as:

$$⊗\varepsilon =\left(\begin{array}{l} ⊗\varepsilon_{x}\\ ⊗\varepsilon_{y}\\ ⊗\gamma_{xy} \end{array}\right)=\left(\begin{array}{c} \frac{\partial ⊗u}{\partial x}\\ \frac{\partial ⊗v}{\partial y}\\ \frac{\partial ⊗v}{\partial x}+\frac{\partial ⊗u}{\partial y} \end{array}\right)$$ (17)

#### 4.2.3 The physics equations with grey variables

The physics equations with grey variables for two-dimension problems can be obtained from the stress-strain relationship determined by experiments:

$$⊗\sigma_{xx}=\frac{⊗E}{1-⊗\mu^{2}}(⊗\varepsilon_{x}+⊗\mu ⊗\varepsilon_{y})$$ (18)

$$⊗\sigma_{yy}=\frac{⊗E}{1-⊗\mu^{2}}(⊗\varepsilon_{y}+⊗\mu ⊗\varepsilon_{x})$$ (19)

$$⊗\sigma_{x\text{y}}=⊗\tau_{\text{x}y}=\frac{⊗E}{2(1+⊗\mu )}⊗\gamma_{xy}$$ (20)

Where ⊗*τ*_x*y*_ is grey shear stress. They can be written in matrix form as:

$$⊗\sigma =⊗D⊗\varepsilon =\left(\begin{array}{l} ⊗\sigma_{xx}\\ ⊗\sigma_{yy}\\ ⊗\tau_{xy} \end{array}\right)=\frac{⊗E}{1-⊗\mu^{2}}\left(\begin{array}{ccc} 1 & ⊗\mu & 0\\ ⊗\mu & 1 & 0\\ 0 & 0 & \frac{1-⊗\mu }{2} \end{array}\right)\left(\begin{array}{c} ⊗\varepsilon_{x}\\ ⊗\varepsilon_{y}\\ ⊗\gamma_{xy} \end{array}\right)$$ (21)

where $⊗D=\frac{⊗E}{1-⊗\mu^{2}}\left(\begin{array}{ccc} 1 & ⊗\mu & 0\\ ⊗\mu & 1 & 0\\ 0 & 0 & \frac{1-⊗\mu }{2} \end{array}\right)$ is elastic matrix with grey variables.

#### 4.2.4 Differential equations with grey variables

By introducing the above geometric equations with grey variables into the physical equations with grey variables, the grey stress represented by grey displacement can be obtained as:

$$⊗\sigma_{xx}=\frac{⊗E}{1-⊗\mu^{2}}(\frac{\partial ⊗u}{\partial x}+⊗\mu \frac{\partial ⊗v}{\partial y})$$ (22)

$$⊗\sigma_{yy}=\frac{⊗E}{1-⊗\mu^{2}}(\frac{\partial ⊗v}{\partial y}+⊗\mu \frac{\partial ⊗u}{\partial x})$$ (23)

$$⊗\sigma_{xy}=\frac{⊗E}{2\left(1+⊗\mu \right)}(\frac{\partial ⊗v}{\partial x}+\frac{\partial ⊗u}{\partial y})$$ (24)

Substituting the above three Eqs (22–24) into the equilibrium Eqs (5 and 6) with grey variables, analytic equations for displacement can be obtained:

$$\frac{⊗E}{1-⊗\mu^{2}}(\frac{\partial^{2}⊗u}{\partial x^{2}}+\frac{1-⊗\mu }{2}\frac{\partial^{2}u}{\partial y^{2}}+\frac{1+⊗\mu }{2}\frac{\partial^{2}⊗v}{\partial x\partial y})+⊗f_{x}=0$$ (25)

$$\frac{⊗E}{1-⊗\mu^{2}}(\frac{\partial^{2}⊗v}{\partial y^{2}}+\frac{1-⊗\mu }{2}\frac{\partial^{2}v}{\partial x^{2}}+\frac{1+⊗\mu }{2}\frac{\partial^{2}⊗u}{\partial x\partial y})+⊗f_{y}=0$$ (26)

With known boundary conditions, the Eqs (25) and (26) can be solved, and the analytical solutions of the grey displacements in the x-direction and y-direction can be calculated. In theory, the grey strain and stress can be obtained through the geometric equations with grey variables and physics equations with grey variables, respectively. But in real engineering cases, because of the complexity of boundary conditions, the equations are challenging to solve. In contrast, discrete numerical analysis is often more efficient. In the following discussion, the FEM is used to study the solution of the differential equations with grey variables. The core content is to convert the continuous infinite degree of freedom problem into a discrete finite degree of freedom problem for solving [26].

## 5. Finite element numerical solution method for differential equations with grey variables

The procedure of conducting the analysis of FEM with grey variables includes: firstly, the studied area was discretized into small elements; and then with the boundary conditions, load conditions and material properties with grey variables, the element stiffness matrix with grey variables, the assembled global stiffness matrix with grey variables and the global equation with grey variables were established. In the above process, the operation was completely carried out according to the operation properties of grey number. Finally, the results of grey displacement, grey stress and grey strain can be calculated. Among them, the establishment of element stiffness matrix with grey variables is the core of the steps [27]. Taking a 4-node rectangular element as an example, the establishment of the element stiffness matrix with grey variables is discussed.

**Fig 3:**
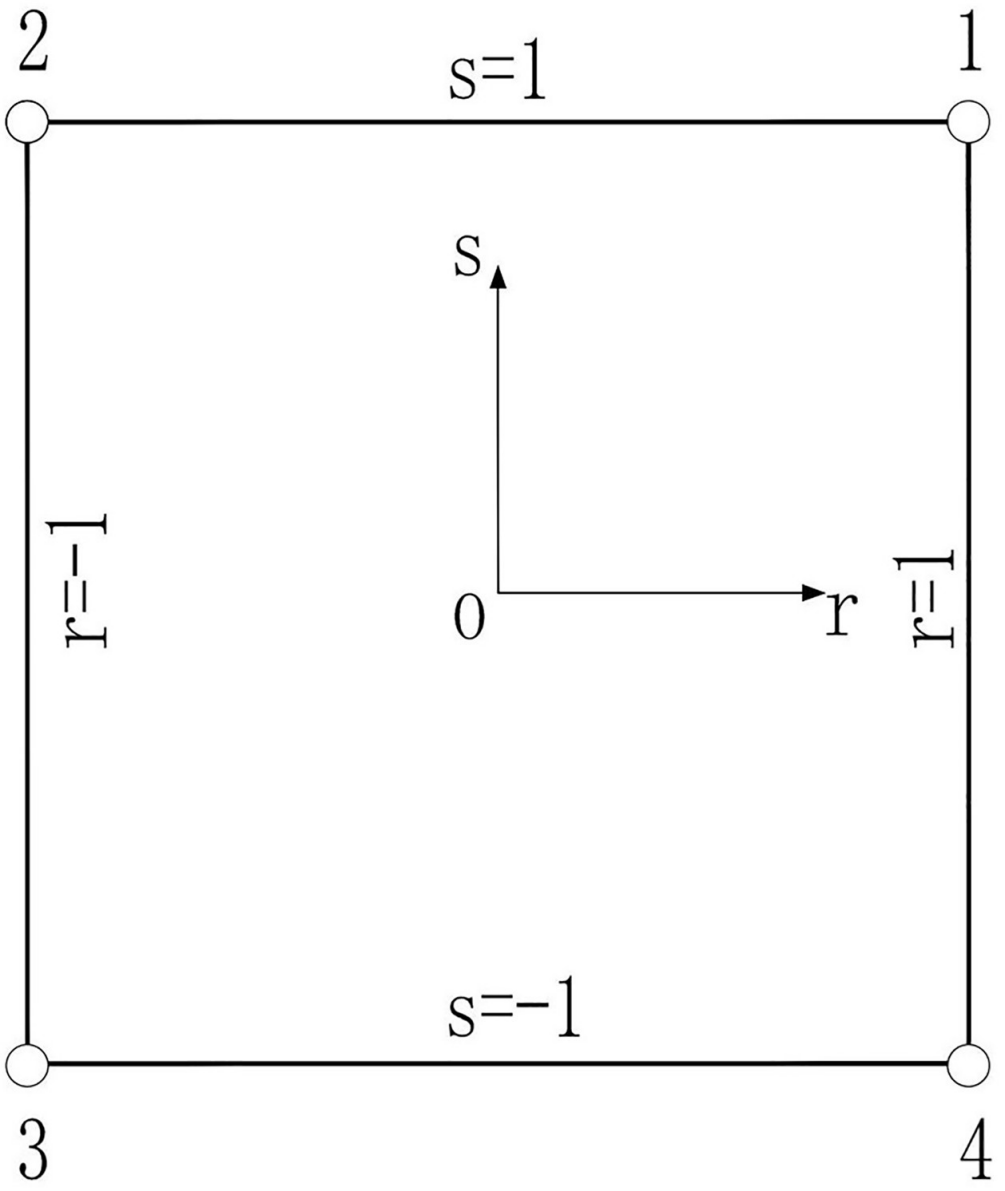
A 4-node rectangular element shape function.

As shown in Fig 3, the shape function [27] of 4-node rectangular element is:

$$\left\{\begin{array}{l} N_{1}=\frac{1}{4}\left(1+r\right)(1+s)\\ N_{2}=\frac{1}{4}\left(1-r\right)(1+s)\\ N_{3}=\frac{1}{4}\left(1-r\right)(1-s)\\ N_{4}=\frac{1}{4}\left(1+r\right)(1-s) \end{array}\right.$$

where r and s are the coordinates in the parent unit. The shape function is used to connect the grey displacement of any point in the element with that of the element node:

$$\left(\begin{array}{l} ⊗u(x,y)\\ ⊗v(x,y) \end{array}\right)=N⊗\delta^{e}=\left(\begin{array}{cccccccc} N_{1} & 0 & N_{2} & 0 & N_{3} & 0 & N_{4} & 0\\ 0 & N_{1} & 0 & N_{2} & 0 & N_{3} & 0 & N_{4} \end{array}\right)\left(\begin{array}{l} ⊗u_{1}\\ ⊗v_{1}\\ ⊗u_{2}\\ ⊗v_{2}\\ ⊗u_{3}\\ ⊗v_{3}\\ ⊗u_{4}\\ ⊗v_{4} \end{array}\right)$$ (27)

where ⊗***δ***^***e***^ = (⊗*u*_1_ ⊗*v*_1_ ⊗*u*_2_ ⊗*v*_2_ ⊗*u*_3_ ⊗*v*_3_ ⊗*u*_4_ ⊗*v*_4_)^*T*^ is grey displacement of the element node, x and y are the coordinates of sub unit, that is, the coordinates in the actual element. It can be obtained from Eqs (17) and (27):

$$⊗\varepsilon =\left(\begin{array}{c} \frac{\partial ⊗u}{\partial x}\\ \frac{\partial ⊗v}{\partial y}\\ \frac{\partial ⊗v}{\partial x}+\frac{\partial ⊗u}{\partial y} \end{array}\right)=\left(\begin{array}{cc} \frac{\partial }{\partial x} & 0\\ 0 & \frac{\partial }{\partial y}\\ \frac{\partial }{\partial y} & \frac{\partial }{\partial x} \end{array}\right)\left(\begin{array}{c} ⊗u\\ ⊗v \end{array}\right)=\left(\begin{array}{cc} \frac{\partial }{\partial x} & 0\\ 0 & \frac{\partial }{\partial y}\\ \frac{\partial }{\partial y} & \frac{\partial }{\partial x} \end{array}\right)N⊗\delta^{e}=B⊗\delta^{e}$$ (28)

where $B=\left(\begin{array}{cc} \frac{\partial }{\partial x} & 0\\ 0 & \frac{\partial }{\partial y}\\ \frac{\partial }{\partial y} & \frac{\partial }{\partial x} \end{array}\right)N=\left(\begin{array}{cccccccc} \frac{\partial N_{1}}{\partial x} & 0 & \frac{\partial N_{2}}{\partial x} & 0 & \frac{\partial N_{3}}{\partial x} & 0 & \frac{\partial N_{4}}{\partial x} & 0\\ 0 & \frac{\partial N_{1}}{\partial y} & 0 & \frac{\partial N_{2}}{\partial y} & 0 & \frac{\partial N_{3}}{\partial y} & 0 & \frac{\partial N_{4}}{\partial y}\\ \frac{\partial N_{1}}{\partial y} & \frac{\partial N_{1}}{\partial x} & \frac{\partial N_{2}}{\partial y} & \frac{\partial N_{2}}{\partial x} & \frac{\partial N_{3}}{\partial y} & \frac{\partial N_{3}}{\partial x} & \frac{\partial N_{4}}{\partial y} & \frac{\partial N_{4}}{\partial x} \end{array}\right)$ is geometric matrix.

According to the theory of FEM and virtual work principle, element stiffness matrix with grey variables can be derived by:

$$⊗K^{e}=\iint B^{T}⊗DB\left|J\right|drds$$ (29)

where $J=\left(\begin{array}{cc} \frac{\partial x}{\partial r} & \frac{\partial y}{\partial r}\\ \frac{\partial x}{\partial s} & \frac{\partial y}{\partial s} \end{array}\right)=\left(\begin{array}{cc} \frac{\partial N_{i}x_{i}}{\partial r} & \frac{\partial N_{i}y_{i}}{\partial r}\\ \frac{\partial N_{i}x_{i}}{\partial s} & \frac{\partial N_{i}y_{i}}{\partial s} \end{array}\right)$ is the Jacobian Matrix.

The element stiffness matrix with grey variables can be then obtained by solving the Eq (29) through Gaussian Integral.

It can be found from the above process that geometric matrix and Jacobian Matrix are variables related to the shape function (***N***) and coordinates (*x*_*i*_, *y*_*i*_). The grey variables only exist in grey elastic matrix. According to grey mathematics [28], the elastic matrix with grey parameters can be calculated by the operation of grey number. The stiffness matrix of each element with grey variables is further calculated. Finally, the global stiffness matrix with grey variables is assembled by the coding method:

$$⊗K=\left(\begin{array}{cccc} ⊗K_{11} & ⊗K_{12} & \cdots & ⊗K_{1n}\\ ⊗K_{21} & ⊗K_{22} & \cdots & ⊗K_{2n}\\ \vdots & \vdots & \vdots & \vdots \\ ⊗K_{n1} & ⊗K_{n2} & \cdots & ⊗K_{nn} \end{array}\right)$$

According to the theory of FEM [26], the global stiffness equation is:

⊗K⊗δ=⊗P (30)

Where ⊗***δ*** = (⊗***u***_1_ ⊗***u***_2_ … ⊗***u***_*n*_)^*T*^ is global node grey displacement array, ⊗***u***_i_ = (⊗*u*_*i*_, ⊗*v*_*i*_)^*T*^ represents the grey displacement of node i in two coordinate directions. ⊗***P*** = (⊗***P***_1_ ⊗***P***_2_ … ⊗***P***_*n*_)^*T*^ is global node grey load array, ⊗***P***_*i*_ = (*X*_*i*_ *Y*_*i*_)^*T*^ represents the grey load of node i in two coordinate directions. From one-to-one correspondence, it can be seen from the relationship between the variables that the physical meaning of global stiffness matrix with grey variables is the force at the i-th node of the element caused by the unit deformation of the j-th degree of freedom of the element. The above process was programmed by Matlab. The calculation of FEM with grey variables was carried out according to the operation properties of grey number.

## 6. Application of the FEM with grey variables and its optimal solution

### 6.1 Analytical solution and classical FEM solution

A deep-buried circular mining tunnel was selected as an example to study the numerical method with grey variables, where a stability evaluation of the surrounding rock is required. The surrounding rock of the tunnel is composed of sandstone and shale, and the geological structure is very complex, with heterogeneity and anisotropy. This geotechnical problem has many grey system characteristics, therefore, the FEM with grey variables is used to solve the problem.

**Fig 4:**
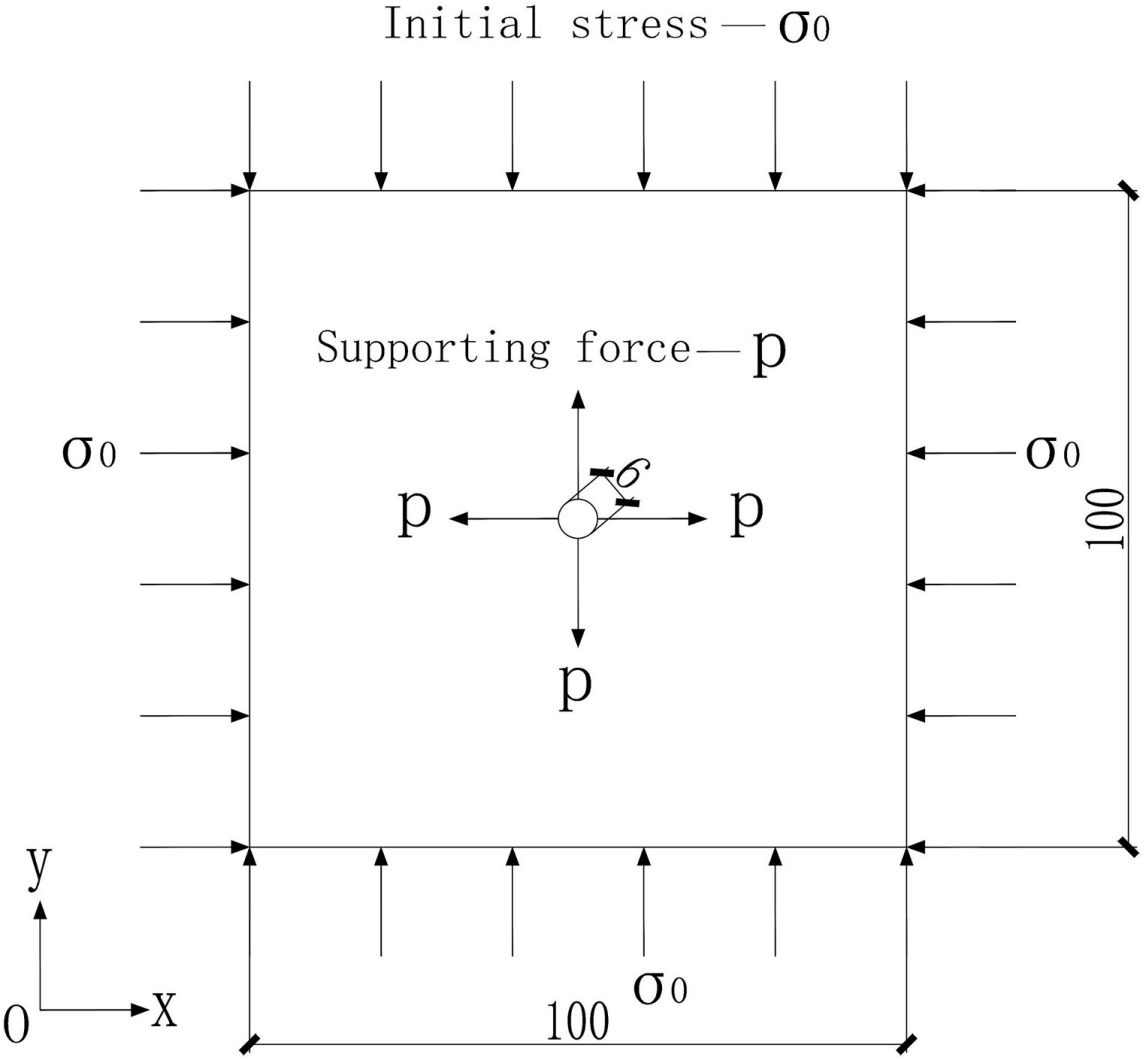
A section of deep-buried elastic circular tunnel.

For clearly study, a section is chosen and it is assumed to be elastic. The deep circular tunnel model with uniform stress field of 100m*100m was built and shown in Fig 4. According to the classical FEM, the parameters and variables are treated to be deterministic, meaning that they have certain numerical values. The parameters in Table 1, are mean values of the measured or experimental ones in the example. However, the determined certain values can hardly cover all the information. In the example, the elastic modulus (E) of surrounding rock mass is 1*10^**8**^ N/m^**2**^, and the Poisson’s ratio (μ) is 0.3. The tunnel’s radius is 3m, and the surrounding area bears uniform initial stress, which is 2*10^**6**^ N/m^**2**^. Supporting force is applied to the inside of the tunnel, the direction is perpendicular to the tangent direction, and the magnitude is 1.06*10^**6**^ N/m^**2**^. The displacement at the boundary of 100m was 0.0011m as calculated [25].

**Table 1:** Parameters, loads and boundary conditions of the example.

Data number	1	2	3	4	5	6	7	8	9	10
E (*10^**8**^ **N/m**^**2**^**)**	0.86	0.88	1.09	1.04	1.06	0.8	1.1	0.98	1.08	1.07
**μ**	0.25	0.28	0.29	0.32	0.35	0.27	0.33	0.34	0.27	0.3
In-situ stress (*10^**6**^ **N/m**^**2**^**)**	1.93	2.05	2.02	1.95	2.07	1.9	2.1	1.92	2.08	2
Supporting force (*10^**6**^ **N/m**^**2**^**)**	1.04	1.10	1.00	1.08	1.07	1.11	1.12	1.03	1.05	1.01
**Boundary displacement (m)**	0.001	0.0011	0.0011	0.0012	0.0010	0.0010	0.0011	0.0012	0.0012	0.0012

**Fig 5:**
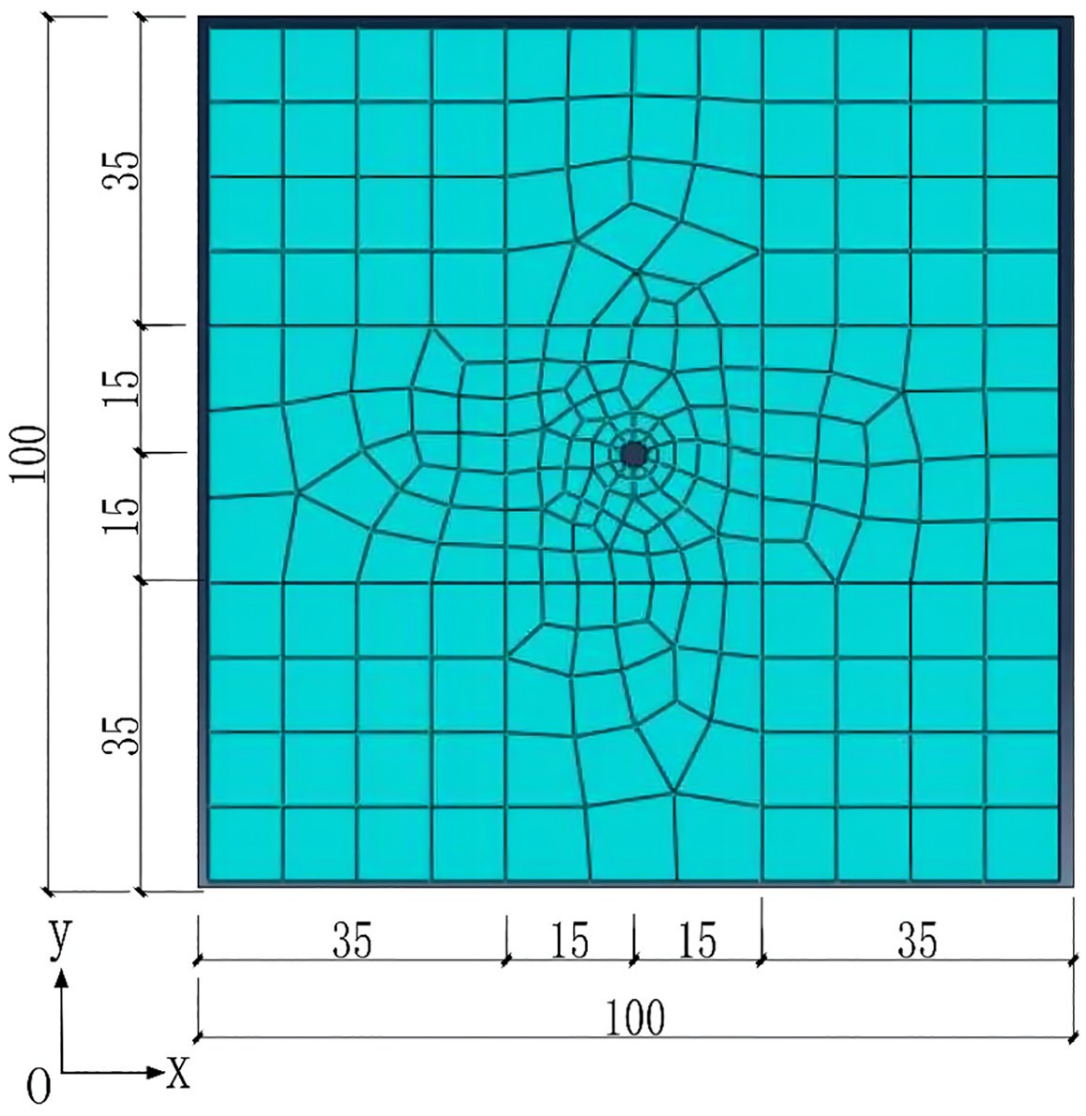
Grid division of the simulation area.

The pre-processing was completed by FEM software Abaqus, which included the establishment of the model, the input of loads and boundary conditions, and the division of the meshes. The divided grid was shown in Fig 5 with 227 elements and 255 nodes in total. The output data from Abaqus (including the node number, node coordinates and load of each node of each element) were used as the input information for the FEM analysis with the grey variables.

When dividing the grid, as the size of the mesh becomes more refined, the numerical solution will approach the exact solution (analytical solution), meaning the numerical solution is getting more accurate. However, small mesh sizes will lead to an excessive increase in the computational cost. Therefore in this study, refined mesh size was only adopted in the key areas in order to meet the accuracy, while for the non-key areas (such as the center distance greater than 10 m in this example), the grid division was relatively rough in order to improve the computational efficiency, as shown in Fig 5.

**Fig 6:**
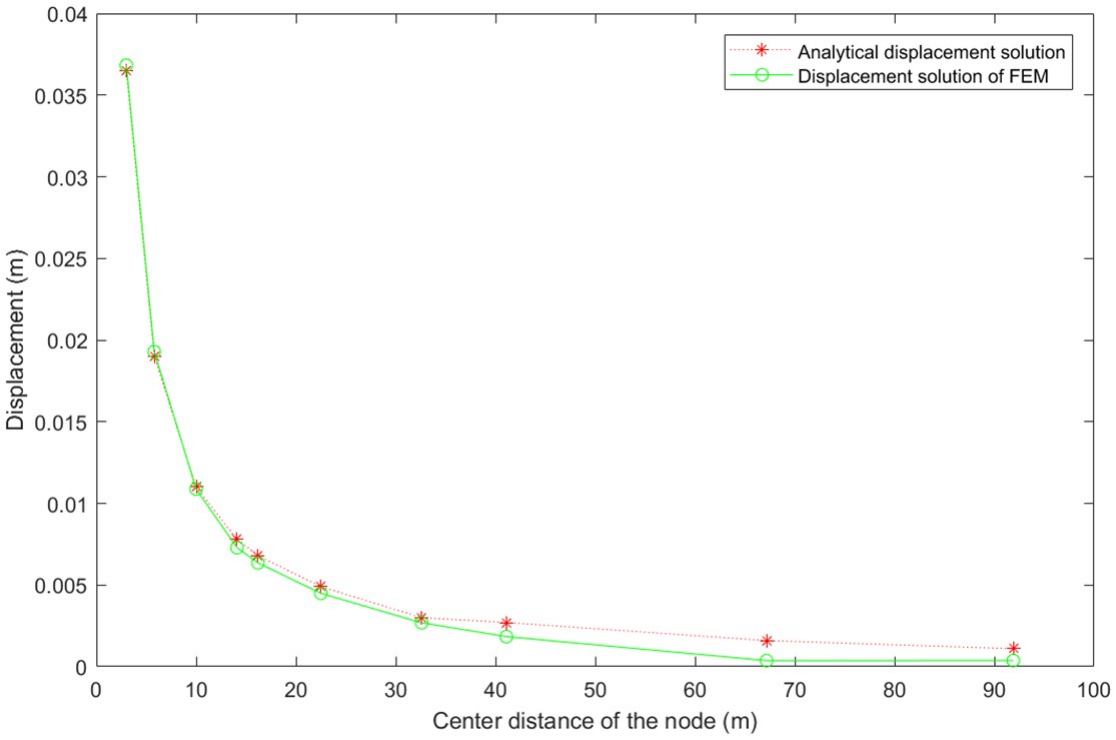
Analytical solution and classical FEM numeric solution-comparison of displacement results.

In order to ensure the correctness of the FEM with grey variables, the classical FEM method was first programmed, and the data pre-processed by Abaqus were used as its input information for calculation. The results were compared with the analytical solution results. Ten representative nodes with different center distances were selected, as shown in Table 2. The comparison of displacement solutions of the nodes was shown in Fig 6. It can be seen from Table 2 that the mesh size increases with the increase of center distance, while the accuracy of numerical solution gradually decreases (i.e. the relative error increases). Regarding the region that we focus on (center distance 3–5 m, where the deformation is critical to tunnel’s support and operation), the accuracy of numerical solution is satisfying, and the relative error is about 1% (i.e. node 69 and node 160).

**Table 2:** Analytical solution and classical FEM numeric solution-comparison of displacement results.

Center distance of the node (m)	Node Number	Displacement (analytical solution)(m)	Displacement (FEM) (m)	Relative error (%)
3	69	0.0368	0.0365	0.82
5.8	160	0.0193	0.019	1.58
10	78	0.0109	0.011	0.91
14.1	225	0.0073	0.0078	6.41
16.2	220	0.0064	0.0068	5.88
22.5	230	0.0045	0.0049	8.16
32.6	110	0.0027	0.003	10
41.1	204	0.0018	0.0027	33.33
67.18	121	0.00037	0.0016	77
91.9	130	0.00038	0.0011	65

**Fig 7:**
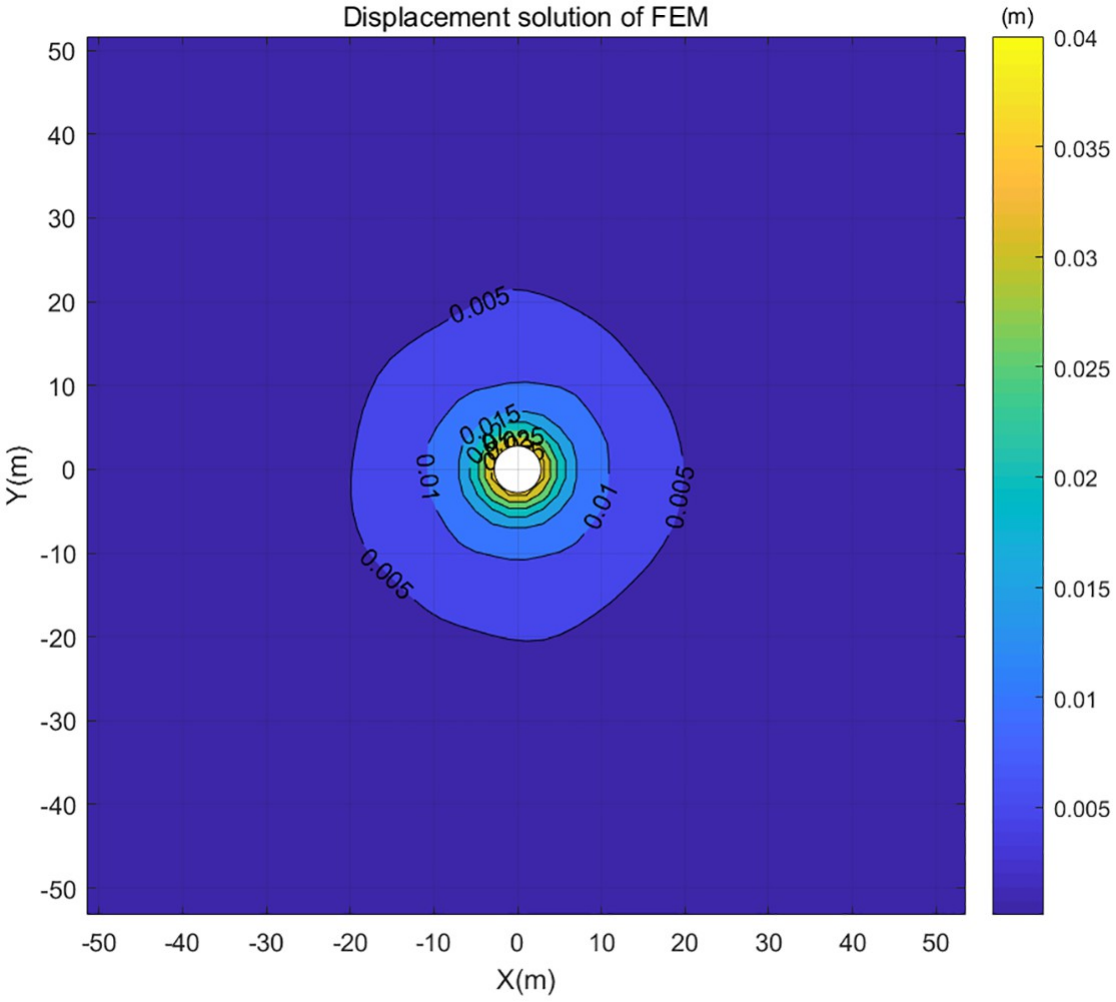
Displacement contour map of classical FEM within 50m center distance.

**Fig 8:**
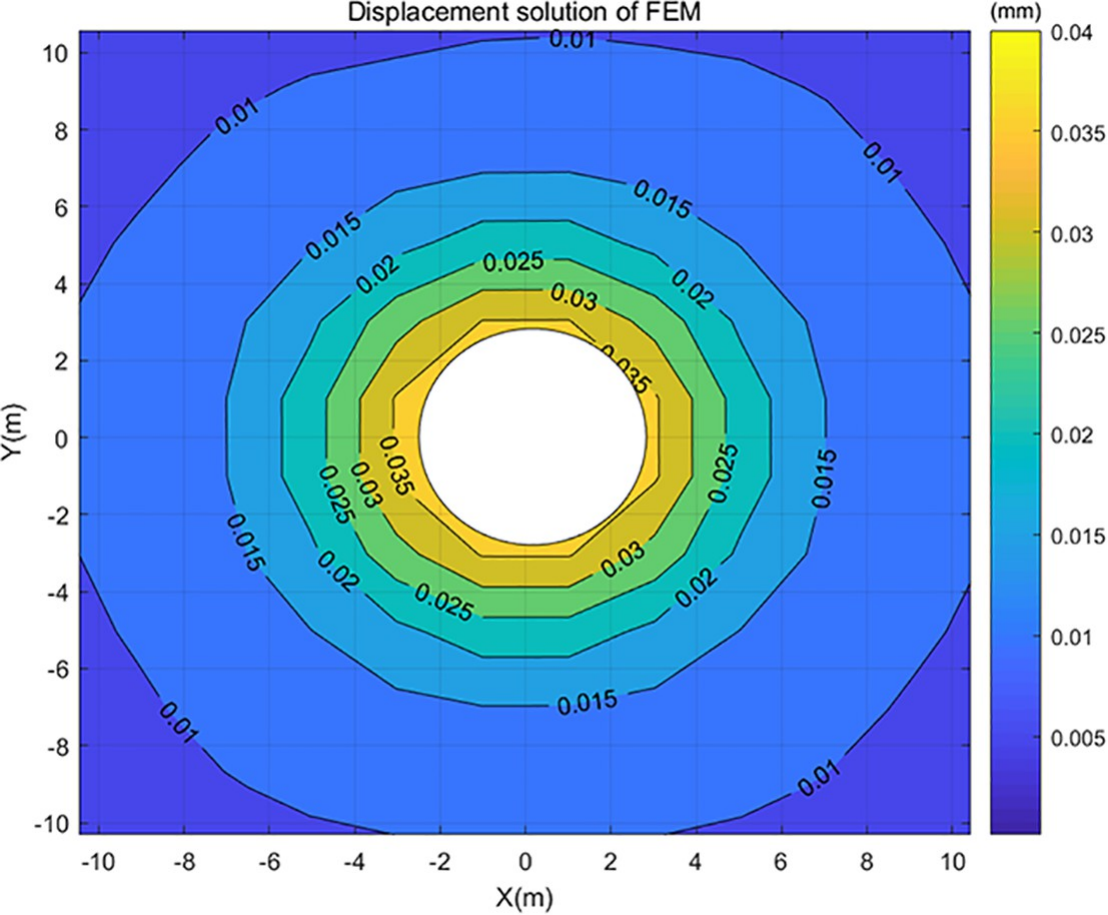
Displacement contour map of classical FEM within 10m center distance.

The calculated displacement by the classical FEM for the meshed grid above was plotted in the centrally symmetric displacement contour maps (Figs 7 and 8). It can be seen from Fig 7 that as the center distance increases, the change of displacement becomes smaller. The displacement of the center distance range of 10m-20m is 0.005m, and the displacement of the center distance ranging from 20m to 100m is about 0m. Therefore, we will focus on studying the displacement within the center distance of 10m, as shown in Fig 8. The related stress and strain were not discussed in this paper.

### 6.2 Determination of optimal solution of FEM with grey variables and its comparison with the analytical solution and classical FEM numerical solution

**Fig 9:**
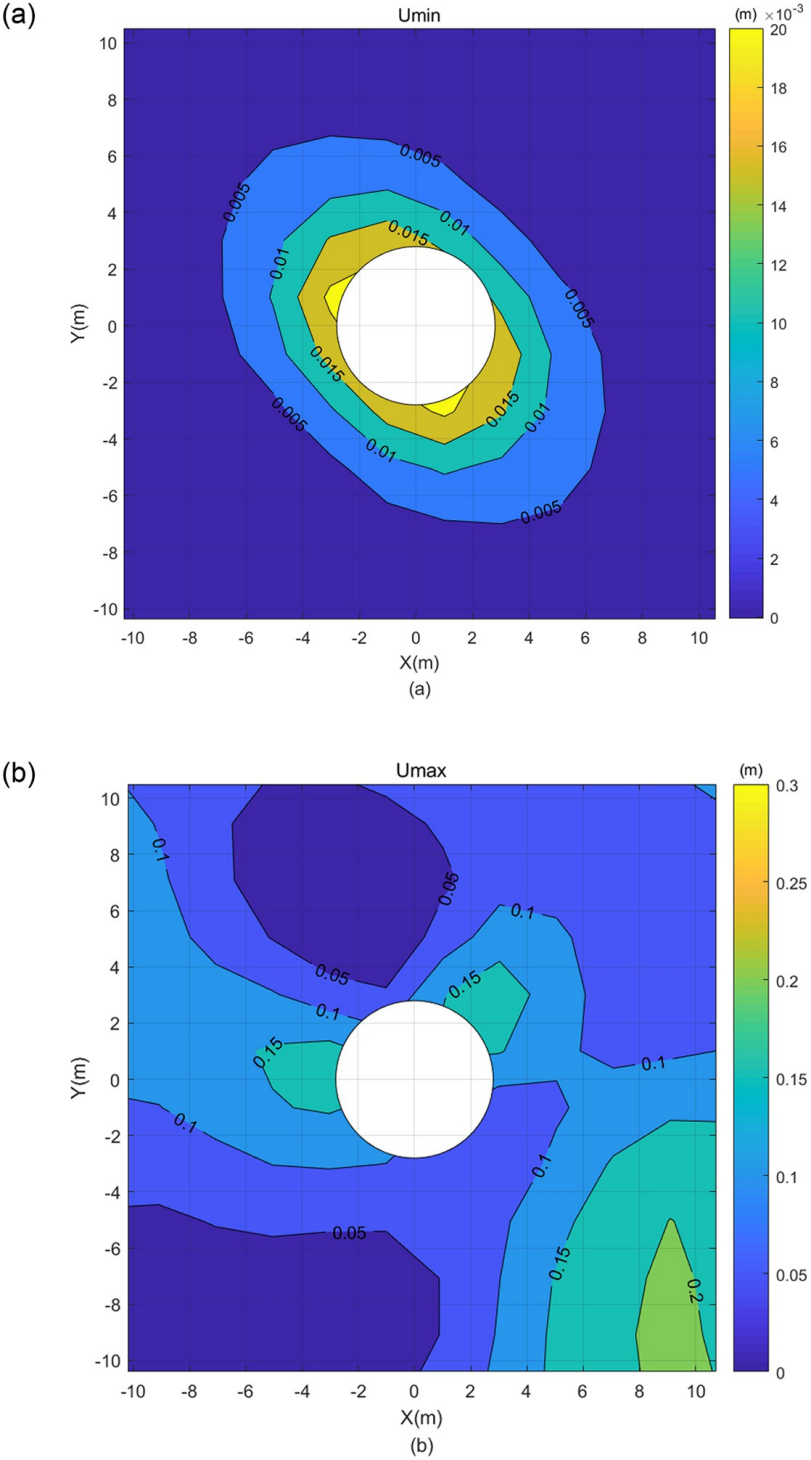
Displacement contour map after calculation with rearranged grey matrix—(a) Umin and (b) Umax.

The determination of parameters and variables in the above example are changed in sense of that the max value and min value of parameters and variables make up the number-covered set of each grey parameters and grey variables, and in this way, all information will be included. So elastic modulus (E) of surrounding rock mass is [0.8*10^8^, 1.1*10^8^] N/m^2^, Poisson’s ratio (μ) is [0.25, 0.35]. The magnitude of uniform initial stress is [1.9*10^6^, 2.1*10^6^] N/m^2^, and the inverse magnitude of support is [1*10^6^, 1.12*10^6^] N/m^2^. The displacement at the boundary of 100m is [0.001, 0.0012] m. The calculation of FEM with grey variables was carried out by the operation properties of grey number, and the following linear equations with the grey variables can be obtained:

$$\left(\begin{array}{cccc} ⊗K_{11} & ⊗K_{12} & \cdots & ⊗K_{1,2n}\\ ⊗K_{21} & ⊗K_{22} & \cdots & ⊗K_{2,2n}\\ \vdots & \vdots & \vdots & \vdots \\ ⊗K_{2n,1} & ⊗K_{2n,2} & \cdots & ⊗K_{2n,2n} \end{array}\right)\left(\begin{array}{c} ⊗u_{1}\\ ⊗v_{1}\\ \vdots \\ ⊗v_{2} \end{array}\right)=\left(\begin{array}{c} ⊗X_{1}\\ ⊗Y_{1}\\ \vdots \\ ⊗Y_{n} \end{array}\right)$$

where $⊗K_{i,j}=\left[\underline{K_{i,j}},\bar{K_{i,j}}\right]$, $⊗X_{i}=\left[\underline{X_{i}},\bar{X_{i}}\right]$, $⊗Y_{i}=\left[\underline{Y_{i}},\bar{Y_{i}}\right]$, $⊗u_{i}=\left[\underline{u_{i}},\bar{u_{i}}\right]$, $⊗v_{i}=\left[\underline{v_{i}},\bar{v_{i}}\right]$ is the grey displacement to solve. The calculation process is computationally intensive, and the results will be distorted if only following the solution method of the interval grey number equations (Fig 9). In this regard, a study on the solution of the grey linear equations was conducted.

It can be seen from Eq (30) that ⊗***δ*** is inversely proportional to ⊗***K*** and proportional to ⊗***P***. The number-covered sets of ⊗***δ*** can be calculated by the following equation:

$$\left\{\begin{array}{l} ⊗\underline{\delta }=(⊗\bar{K}{)}^{-1}⊗\underline{P}\\ ⊗\bar{\delta }=(⊗\underline{K}{)}^{-1}⊗\bar{P} \end{array}\right.$$ (31)

The Eq (29) shows that when the element stiffness matrix with grey variables is calculated, only the elastic matrix ⊗***D*** contains grey parameters. When ⊗***D*** is calculated, according to the operation properties of grey number, the lower bound of each element obtained is less than the upper bound. However, when the element stiffness matrix with grey variables is calculated, the lower bound of some elements becomes greater than the upper bound. The load matrix with grey variables also has the same problem. By rearranging the elements of the stiffness matrix with grey variables and the load matrix with grey variables with the rule that the lower bound is smaller than the upper bound, and calculating with Eq (31), the results can be derived as the displacement contour map in Fig 9.

**Fig 10:**
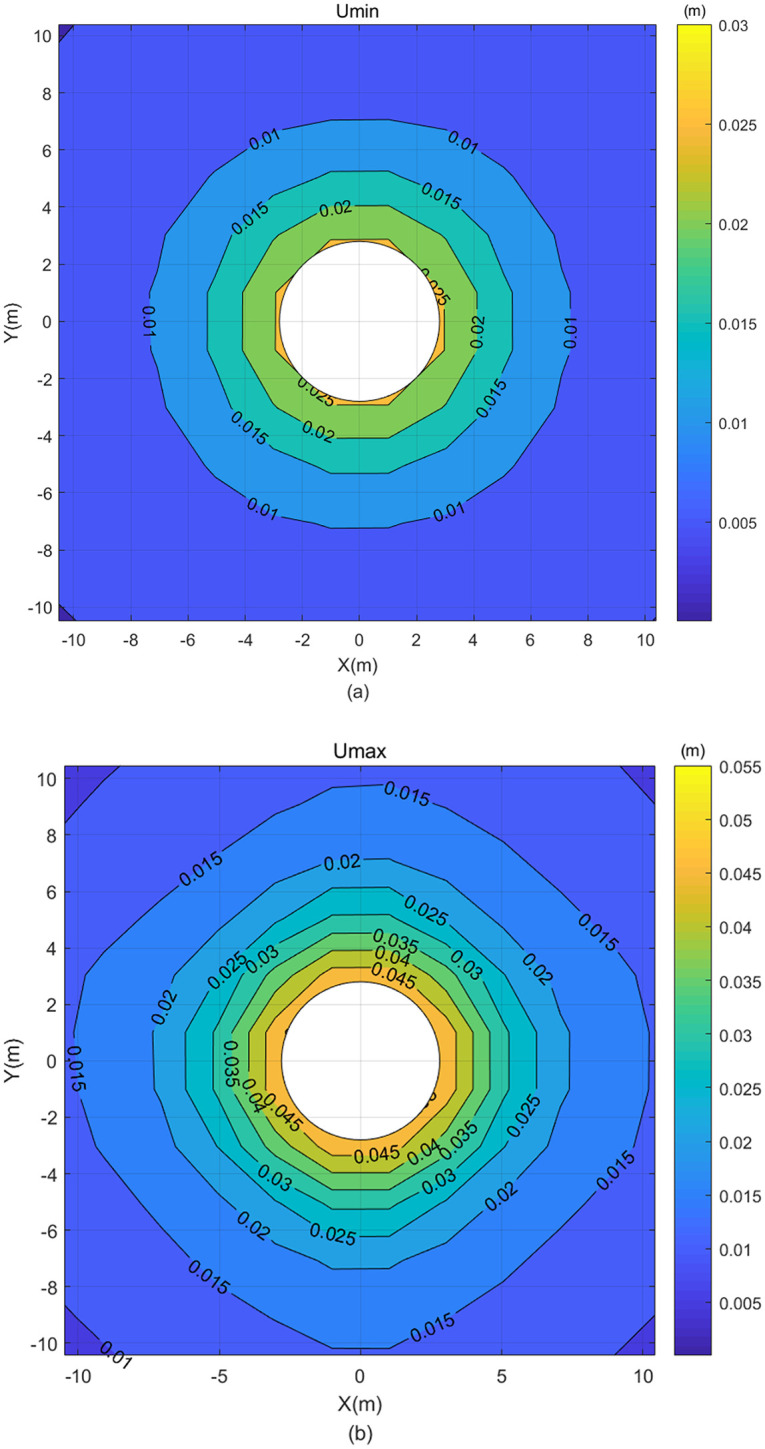
Displacement contour map calculated by Eq (31) without rearranging the grey matrix—(a) Umin and (b) Umax.

It can be seen from the comparison in Fig 9 that most of the displacement of Umax is greater than that of Umin, but the displacement distribution of Umax is irregular making its reference value limited. Although the displacement distribution of Umin is more regular, its reference value is also poor because it is contrast to the above result that the displacement is inversely proportional to center distance. This is because after the element stiffness matrix with grey variables and the load matrix with grey variables were rearranged, the originally uniform and regular solution area became irregular. Therefore in this study, after calculating the elastic matrix **⊗*D*** (in which the lower bound of each element in ⊗***D*** is less than the upper bound) through the operation properties of grey number, and further calculating the element stiffness matrix with grey variables (some lower bounds in the stiffness matrix of the grey element may be greater than the relative upper bounds), the element stiffness matrix with grey variables and the load matrix with grey variables were not rearranged. The obtained displacement solutions were plotted in Fig 10.

It can be seen from Fig 10 that all the displacement of Umax is greater than Umin, and the grey displacement distribution is regular. Comparing with the classical FEM, it can be found that the displacement results of the classical FEM are included within the chaos (defined in Definition 3.2 (d)) of grey displacements of the FEM with grey variables, which implies the model from Eq (31) is more reasonable and has more information considered than the classical FEM. For example, when the center distance is 4m, the displacement of the classical FEM is 0.025m, and the displacement of FEM with grey variables is [0.02, 0.04] m.

**Fig 11:**
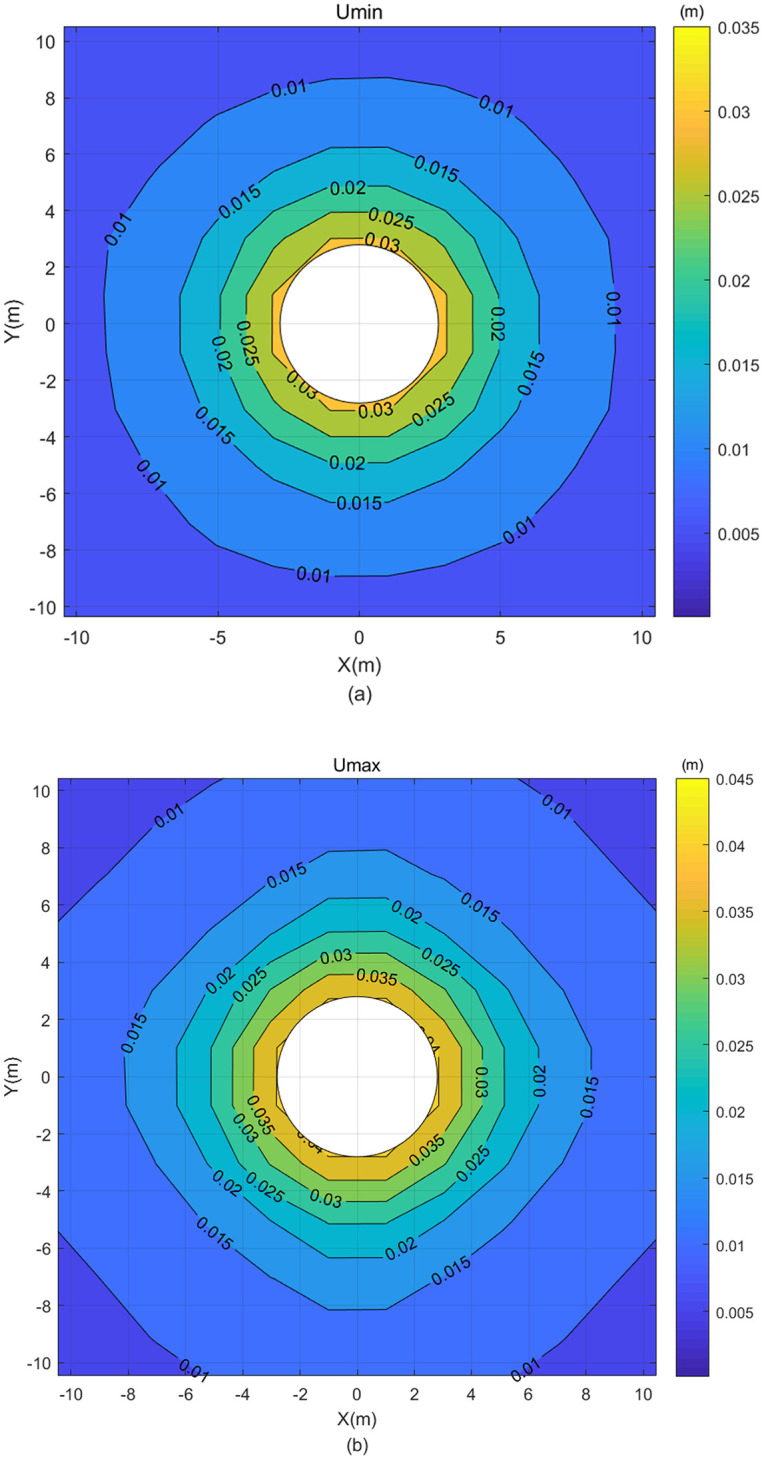
Displacement contour map calculated by Eq (32) without rearranging the grey matrix—(a) Umin and (b) Umax.

Similarly, the other solution can be calculated from Eq (32) (in Fig 11) when the elastic matrix ⊗***D*** (in which the lower bound of each element in ⊗***D*** is less than the upper bound) is calculated through the operation properties of grey number, and the element stiffness matrix with grey variables and the load matrix with grey variables are not rearranged:

$$\left\{\begin{array}{c} ⊗\underline{\delta }={\left(⊗\underline{K}\right)}^{-1}⊗\underline{P}\\ ⊗\bar{\delta }={\left(⊗\bar{K}\right)}^{-1}⊗\bar{P} \end{array}\right.$$ (32)

It can be seen from Fig 11 that the Umax is greater than the Umin, the distribution is regular, and the displacement results of the classical FEM are also included within the chaos of grey displacements of the FEM with grey variables, making the results calculated by Eq (32) correct and reasonable. Furthermore, the chaos of grey displacements calculated by Eq (32) are included within those calculated by Eq (31). Therefore, Eq (31) is more advantageous than Eq (32), and it provides the optimal solution of FEM with grey variables in this study.

**Fig 12:**
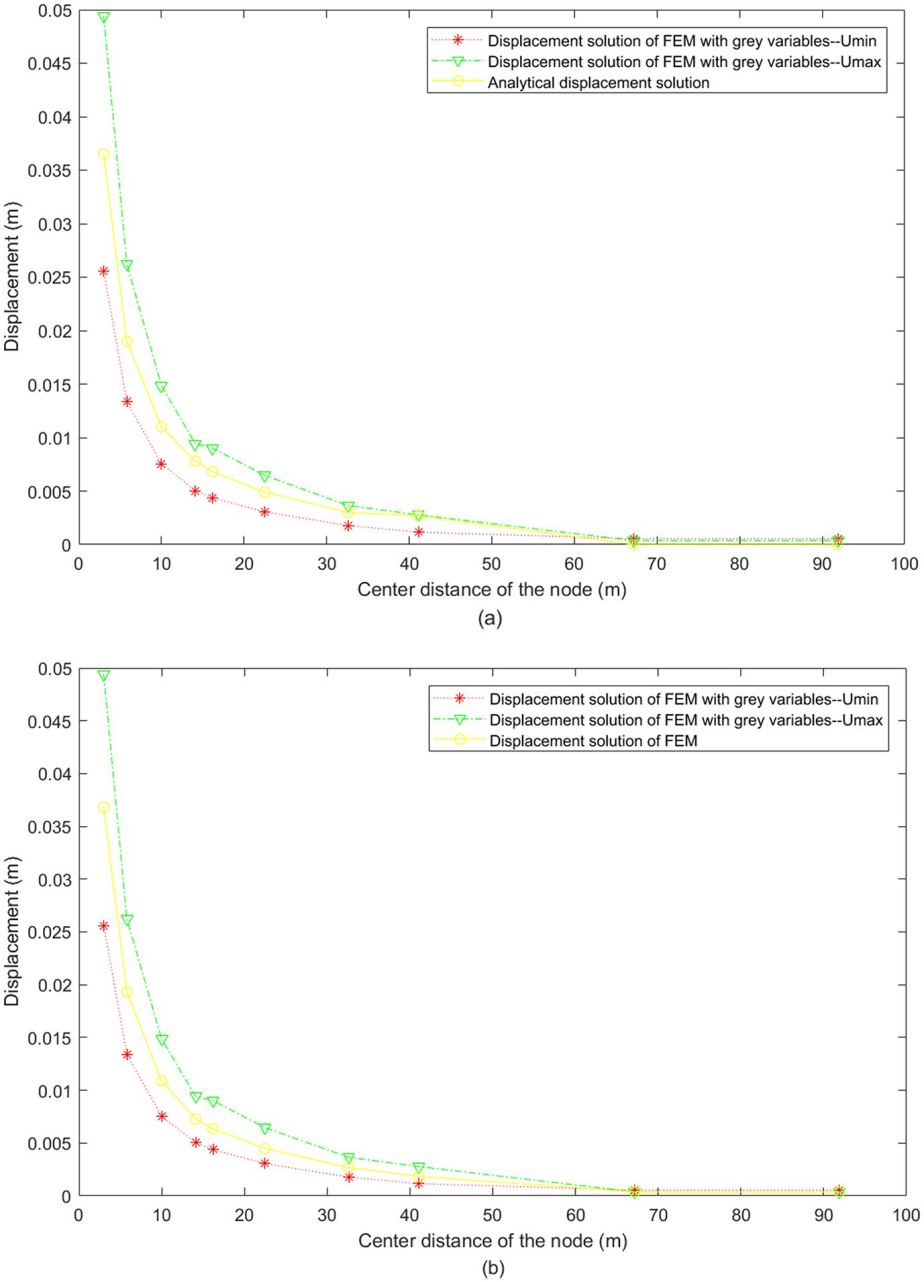
Comparison of FEM with grey variables (a) with analytical solution and (b) with classical FEM solution.

The displacement solution (chaos of grey displacement) of FEM with grey variables was compared with the analytical displacement solution and the classical FEM displacement solution, respectively. Ten nodes were selected as mentioned above, and the displacement comparison was plotted in Fig 12. It can be seen that the analytical displacement solution and the classical FEM displacement solution are both included in the chaos of grey displacement solution of FEM with grey variables, indicating that the FEM with grey variables is reasonable. Moreover, bigger chaos of grey displacement is obtained when center distance of the node is smaller (meaning a greater grid accuracy, i.e. node 69 and node 160). This is because smaller center distance corresponds to bigger stress, and further causes more complex deformation situation and larger chaos of grey displacement. It is worth pointing out that greater chaos of grey displacement does not represent greater calculation error, on the contrary it means the results are more safe by including more uncertainty information which is missed in the classical FEM.

## 7. Conclusions

1. A new idea of numerical simulation of geotechnical problems is proposed, i.e. constructing the numerical model with grey system theory. Based on the grey system theory, this paper analyzed the grey properties, grey parameters and grey variables of rock and soil system, established the basic equilibrium equations and the FEM equations with grey variables, and used the operation properties of grey number to solve the FEM equations with grey variables.
2. An example of a deep-buried circular mining tunnel was executed, and the displacement solution of FEM with grey variables was obtained and compared with the analytical displacement solution and classical FEM displacement solution. The comparison results show that both the analytical displacement solution and classical FEM displacement solution were included in the chaos of grey displacement calculated by FEM with grey variables, and the accuracy of calculation results was improved with more refined mesh size. This demonstrates that the results of the proposed grey numerical model contain more information (i.e. the bigger chaos of the displacement), and the potential danger of losing some information in case of applying the classical numerical model can be avoided. So the proposed grey numerical model is more reliable when dealing with real geotechnical problems.
3. The chaos of grey displacement of FEM with grey variables increases as the center distance decreases, due to the fact that the smaller center distance of the node (meaning higher the grid accuracy, i.e. node 69 and node 160) is, the bigger stress and the more complex deformation situation is. A larger chaos of grey displacement suggests that the result be more reliable, making the proposed model capable of handling the complexity in real cases.
